# Prey species and abundance affect growth and photosynthetic performance of the polyphagous sea slug *Elysia crispata*

**DOI:** 10.1098/rsos.230810

**Published:** 2023-08-30

**Authors:** Paulo Cartaxana, Luca Morelli, Elena Cassin, Vesa Havurinne, Miguel Cabral, Sónia Cruz

**Affiliations:** ECOMARE – Laboratory for Innovation and Sustainability of Marine Biological Resources, CESAM – Centre for Environmental and Marine Studies, Department of Biology, University of Aveiro, Aveiro 3810-193, Portugal

**Keywords:** *Acetabularia acetabulum*, *Bryopsis plumosa*, chloroplast, kleptoplast retention time, Sacoglossa, starvation tolerance

## Abstract

Some sacoglossan sea slugs steal functional macroalgal chloroplasts (kleptoplasts). In this study, we investigated the effects of algal prey species and abundance on the growth and photosynthetic capacity of the tropical polyphagous sea slug *Elysia crispata*. Recently hatched sea slugs fed and acquired chloroplasts from the macroalga *Bryopsis plumosa,* but not from *Acetabularia acetabulum*. However, adult sea slugs were able to switch diet to *A. acetabulum*, rapidly replacing the great majority of the original kleptoplasts. When fed with *B. plumosa*, higher feeding frequency resulted in significantly higher growth and kleptoplast photosynthetic yield, as well as a slower relative decrease in these parameters upon starvation. Longevity of *A. acetabulum*-derived chloroplasts in *E. crispata* was over twofold that of *B. plumosa*. Furthermore, significantly lower relative weight loss under starvation was observed in sea slugs previously fed on *A. acetabulum* than on *B. plumosa*. This study shows that functionality and longevity of kleptoplasts in photosynthetic sea slugs depend on the origin of the plastids. Furthermore, we have identified *A. acetabulum* as a donor of photosynthetically efficient chloroplasts common to highly specialized monophagous and polyphagous sea slugs capable of long-term retention, which opens new experimental routes to unravel the unsolved mysteries of kleptoplasty.

## 1. Introduction

Photosynthesis is not limited to plants, algae and cyanobacteria. Animals, like corals, usually acquire photosynthetic properties via symbiosis, where the animal host lives in mutualistic harmony with an intact algal or cyanobacterial symbiont [1]. Certain marine invertebrates have evolved a different approach that achieves a similar, yet very distinct, outcome in terms of their energy metabolism. Indeed, so-called kleptoplastic invertebrates take only the photosynthetic cell organelles, chloroplasts, from their algal prey, and incorporate them as kleptoplasts in their own cells [2,3]. By far the most studied kleptoplastic animals belong to the Sacoglossa, a group of sea slugs that contains multiple species capable of harbouring photosynthetically active kleptoplasts for weeks and months [4]. This is no small feat for a sea slug that does not have access to the genetic material of the algal nucleus containing most of the genes involved in the day-to-day regulation and maintenance of chloroplasts.

Some of the studies on monophagous sacoglossans like *Elysia timida* and its sister species *Elysia cornigera* have shown that while both feed on a specific alga, *Acetabularia acetabulum* (Linnaeus) P.C. Silva, 1952, only *E. timida* is able to retain long-term functional kleptoplasts [5,6]. This indicates that even if the original chloroplasts are the same, not all sacoglossan sea slugs are equal in their capacity for kleptoplasty. Similarly, the polyphagous sea slug *Elysia crispata* Mörch, 1863 can retain long-term kleptoplasts, while other sacoglossan sea slugs (e.g. *Elysia patina*, *Elysia rufescens*) using similar algal food sources are unable to maintain the foreign organelles for more than a week [7]. Experimental studies on kleptoplast functionality in polyphagous sea slug species fed with specific algal chloroplast donors are scarce. An exception is the work by Curtis *et al*. [8] that compares the longevity of the kleptoplasts in *E. crispata* fed exclusively with either *Bryopsis plumosa* (Hudson) C. Agardh, 1823 or *Penicillus lamouroxii*. The authors found that specimens fed on *P. lamouroxii* maintain photosynthetic activity four weeks longer than those fed with *B. plumosa*. Therefore, it is reasonable to assume that the capacity for long-term maintenance of functional chloroplasts in sacoglossan sea slugs results from the combination of mostly uncharacterized mechanisms within the animal host cells with the inherent robustness of the original chloroplasts [9].

The record for the longest retention of kleptoplasts, approximately 9–10 months, is held by *Elysia chlorotica*, a sea slug capable of very effectively incorporating and maintaining the functional plastids while feeding on a single alga, *Vaucheria litorea,* with innately robust chloroplasts [10,11]. The second longest kleptoplast retention time is somewhere between *E. timida* and the polyphagous sacoglossans *Plakobranchus ocellatus* and *E. crispata* [8,12,13]. The comparison between studies is sometimes hindered by the variability in wild-collected specimens and/or differences in laboratory conditions used to assess kleptoplast longevity [14]. Light intensity at which the sea slugs are reared is particularly relevant, with higher irradiances leading to significantly reduced chloroplast functionality [15,16]. Surprisingly, to our knowledge, no reports exist in the literature on kleptoplast longevity and starvation tolerance of polyphagous sea slug species that have been specifically fed with some of the known high-performance chloroplast containing prey algae of the monophagous species *E. chlorotica* or *E. timida*.

The tropical sea slug *E. crispata* (sometimes referred to as its junior synonym *Elysia clarki*) is a large sacoglossan with a distribution ranging from the northern coast of South America, throughout the Caribbean and up the Florida Keys [17]. The species can be reared in captivity through its whole life cycle by feeding on only one alga, *B. plumosa*, but adult specimens are known to feed on a wide variety of algae in the wild [18,19]. In this study, we investigated the effects of algal prey species (*B. plumosa* or *A. acetabulum*) on growth, starvation tolerance, photosynthetic capacity and kleptoplast longevity in the sea slug *E. crispata*.

## 2. Methods

### 2.1. Rearing of *Elysia crispata* and algal culturing

*Elysia crispata* sea slugs originally collected in Florida (USA) and purchased from TMC Iberia (Lisbon, Portugal) were reared in 150 l recirculated life support systems (LSS) operated with artificial seawater (ASW) at 25°C and a salinity of 35‰. The photoperiod was set to 12 h light : 12 h dark, with a photon scalar irradiance of 60–80 µmol photons m^−2^ s^−1^ being provided by T5 fluorescent lamps. Irradiance was measured in the water with a Spherical Micro Quantum Sensor and a ULM-500 Universal Light Meter (Walz, Effeltrich, Germany).

Macroalgae were cultivated separately to feed *E. crispata* sea slugs. The green alga *B*. *plumosa*, acquired from Kobe University Macroalgal Culture Collection (KU-0990, KUMACC, Japan), was grown in 2 l flasks with f/2 medium (without silica) and constant aeration at 20°C and an irradiance of 60–80 µmol photons m^−2^ s^−1^ provided by LED lamps (Valoya 35 W, spectrum NS12). The green alga *A. acetabulum* (strain DI1 originally isolated by Diedrik Menzel) was cultivated essentially as described earlier by Havurinne and Tyystjärvi [20] in 3–20 l transparent plastic boxes filled with f/2 medium without aeration under the same lamps as *B. plumosa*. The photon scalar irradiance was set to 40 µmol photons m^−2^ s^−1^ for *A. acetabulum*. Both *B. plumosa* and *A. acetabulum* were grown in the same 12 : 12 h photoperiod as *E. crispata*.

### 2.2. Effects of macroalgal prey species on chloroplast acquisition

Egg masses from *E. crispata* specimens laid at the walls of the LSS were collected with a scalpel and transferred to individual 250 ml Erlenmeyer flasks with autoclaved ASW until hatching. Two to three days after the onset of hatching, the sea slugs from 10 different egg masses were provided with either *B. plumosa* or *A. acetabulum* (five replicates per alga). At 20 days post-hatching, the sea slugs were moved to aerated 5 l plastic transparent boxes filled with autoclaved ASW (water was changed once per week) and fed once per week. Both egg masses and juveniles were reared under the same temperature, salinity and light conditions described above for adults. Because the recently hatched sea slugs offered *A. acetabulum* did not feed and died, 3–3.5-month-old sea slugs feeding on *B. plumosa* were again offered *A. acetabulum* to observe if the animals could switch diet. Five sea slugs offered 120 mg of *A. acetabulum*, provided for 10 days, were snap frozen in liquid nitrogen, freeze-dried and stored at –80°C. Five sea slugs fed on *B. plumosa* were sampled similarly and used for pigment composition analysis (see below).

### 2.3. Effects of macroalgal food availability on growth and kleptoplast photosynthesis

A floating tray with wells (56 mm diameter × 60 mm depth) was placed floating in a LSS with the same temperature, salinity, photoperiod and irradiance described above for the rearing of *E. crispata*. The bottom of the wells was made of a 0.5 mm mesh to allow water exchange. A recirculating water pump was placed below the experimental tray to increase water renewal inside the wells. Thirty 2–2.5-month-old sea slugs were randomly placed in individual wells. Three different feeding treatments with *B. plumosa* (10 replicates per treatment) were performed: (i) fed every 2 days, (ii) fed once per week, and (iii) fed once every two weeks. Initial weights of individual sea slugs in three treatments were similar: 35 ± 10 mg, 35 ± 8 mg and 36 ± 9 mg (mean ± s.d.) for (i), (ii) and (iii), respectively. All sea slugs were initially supplied with 50 mg fresh weight of *B. plumosa* until day 28, and with 100 mg fresh weight of algae from day 28 until the end of the experimental period (58 days), to avoid the bigger animals exhausting their food. Regardless of the feeding treatment, sea slugs were able to feed for 48 h, after which the algae were removed from the wells. At this point, new algae were added in treatment (i), while in treatments (ii) and (iii) the animals were provided with food only in the next feeding date (5 and 12 days later, respectively).

### 2.4. Effects of previous feeding history on sea slug weight and photosynthesis during starvation

Sea slugs from the previous feeding experiment with *B. plumosa* were starved for 45 days in individual wells in the LSS described above. After this period, no measurable variable chlorophyll (Chl) fluorescence could be detected in most of the specimens (see below *Photosynthetic performance*). Ten additional 3–3.5-month-old sea slugs, which were previously fed once per week with *B. plumosa* (48 h of feeding followed by 5 days of starvation), were placed in individual wells in the LSS and offered 120 mg of *A. acetabulum* provided for 10 days. This period of time was sufficient for the replacement of the great majority of the original *B. plumosa* chloroplasts by *A. acetabulum* chloroplasts, as verified by pigment analysis. All the adult sea slugs that were offered *A. acetabulum* were able to switch diet and, after feeding on the new food source, were starved for 75 days.

### 2.5. Sea slug weight

The sea slugs fed on *B. plumosa* were weighed at the start of the feeding experiment and then every two weeks during the 58 days experimental period. To determine the fresh weight, the animals were individually transferred to a 200 μm nylon mesh sieve that was placed on top of tissue paper to remove the excess of water, weighed and returned to their individual wells in the LSS. During the starvation experiment, sea slugs fed on *B. plumosa* were weighed initially every 6 days until day 12, then every 3 days until the end of the experimental period (45 days). Sea slugs fed on *A. acetabulum* were weighed every 5 days during the feeding period, once a week until day 49 of starvation, every 4 days between day 49 and day 69 and every 3 days until the end of the experimental period (75 days). The individual daily relative loss of fresh weight during starvation was calculated as (1 − (*W*_f_/*W*_0_))/*t* × 100, where *W*_f_ and *W*_0_ indicate the sea slugs' fresh weights at the end and at the beginning of the starvation period, respectively, and *t* is the duration in days of the starvation period.

### 2.6. Photosynthetic performance

Chl fluorescence measurements were performed using an Imaging-PAM fluorometer, M-series, Mini-version (Walz, Effeltrich, Germany), equipped with an IMAG-K7 camera. Prior to each measurement, sea slugs were dark-adapted for 20 min. Specimens were then placed, one at a time, on a watch glass with a minimal amount of water to restrain their movement. A saturating-light pulse was applied, and the minimum and maximum fluorescence levels were recorded (*F*_o_ and *F*_m_, respectively) (electronic supplementary material, figure S1). Numerical values of variable Chl *a* fluorescence parameters were obtained from areas of interest (AOI) in the digital images using the ImagingWin software (Walz, Effeltrich, Germany). The maximum quantum yield of photosystem (PS) II (*F*_v_/*F*_m_) was calculated as (*F*_m_ – *F*_o_)/*F*_m_ [21]. The parameter *F*_v_/*F*_m_ was used as an indicator of photosynthetic performance. During the feeding experiment, *F*_v_/*F*_m_ was measured at the beginning of the experiment and at day 56, immediately before feeding. *F*_v_/*F*_m_ was measured again 2 days after macroalgal food was provided to all the sea slugs (day 58). In the starvation experiments, *F*_v_/*F*_m_ was measured on the same dates as the weight measurements. The individual daily relative loss of *F*_v_/*F*_m_ during starvation was calculated as (1 − (*F*_f_/*F*_i_))/*t* × 100, where *F*_f_ and *F*_i_ indicate the sea slugs’ *F*_v_/*F*_m_ at the end and at the beginning of the starvation period, respectively, and *t* is the duration in days of the starvation period.

### 2.7. Pigment analysis

Pigment analysis was performed by high performance liquid chromatography (HPLC) as described in detail by Cruz *et al*. [22]. Briefly, freeze-dried sea slugs were extracted for 20 min in 95% cold buffered methanol (2% ammonium acetate) at –20°C, after 1 min sonication. After filtration, the extracts were injected into a Prominence-i LC 2030C HPLC system (Shimadzu, Kyoto, Japan) with a photodiode array detector. Chromatographic separation was carried out using a Supelcosil C18 column (dimensions: 250 × 4.6 mm; particle size: 5 µm; Sigma-Aldrich, St Louis, MO, USA) for reverse phase chromatography. Photosynthetic pigments were identified from retention times and absorption spectra, in comparison with pure crystalline standards from DHI (Hørsolm, Denmark).

### 2.8. Statistical analysis

To assess if sea slug fresh weight variation over time was dependent on macroalgal food availability, an aligned rank test (ART) was performed, as the assumptions of the factorial mixed ANOVA were not satisfied. To test the effects of macroalgal food availability on maximum photosynthetic yield (*F*_v_/*F*_m_), the three feeding treatments were compared on day 0, day 56 and day 58. For that purpose, a Kruskal–Wallis test was performed, followed by Dunn's tests for pairwise comparisons whenever a significant result was observed. An ART was implemented to evaluate the effects of the previous feeding history on changes of sea slug relative weight and *F*_v_/*F*_m_ during starvation. The average daily relative loss of fresh weight and *F*_v_/*F*_m_ was compared between sea slugs that were previously fed with the macroalgae *B. plumosa* or *A. acetabulum* by applying Mann–Whitney *U* tests. Lastly, to compare the *F*_v_/*F*_m_ of the sea slugs before and after being fed with *A. acetabulum* a Student's *t*-test for paired samples was performed. The significance level was considered as 0.05 and all *p*-values obtained by multiple comparisons were adjusted using Bonferroni's correction. Statistical analyses were performed using RStudio (v. 4.0.2).

## 3. Results

### 3.1. Effects of macroalgal prey species on chloroplast acquisition

**Figure 1.**
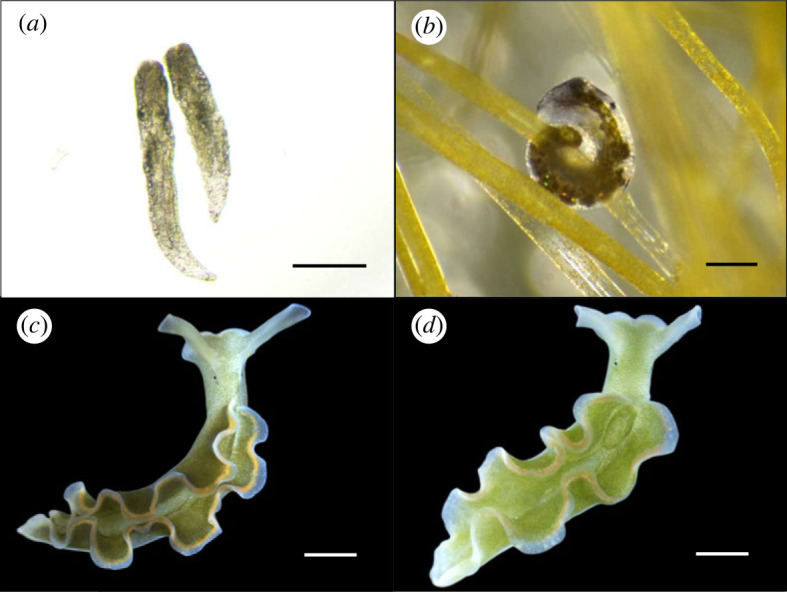
Recently hatched *E. crispata* before feeding (*a*) and while feeding on the macroalga *B. plumosa* (*b*) from which they steal functional chloroplasts (kleptoplasts). Upon hatching, sea slugs were unable to feed directly on the macroalga *A. acetabulum*. However, three-month-old sea slugs feeding on *B. plumosa* (*c*) were able to switch diet to *A. acetabulum*, replacing the original chloroplasts within 5–10 days (*d*). Photo (*a*) was taken in an inverted microscope (DMi1, Leica Microsystems, Wetzlar, Germany), while (*b*), (*c*) and (*d*) were obtained using a digital microscope (DMS-300, Leica Microsystems). Scale bars represent 250 µm in (*a*) and (*b*) and 2 mm in (*c*) and (*d*).

Recently hatched *E. crispata* sea slugs offered the macroalga *B. plumosa* were able to feed and acquire the algal chloroplasts (figure 1*a*,*b*). Juveniles hatching from all five egg masses were able to grow and attained a dark green coloration upon feeding on *B. plumosa* (figure 1*c*). On the contrary, none of the recently hatched sea slugs offered *A. acetabulum* could feed and retain the chloroplasts and died within a few days after hatching. However, after approximately three months of feeding on *B. plumosa*, animals offered *A. acetabulum* were able to switch diet. Within 5 to 10 days, the coloration of these animals changed, acquiring a lighter green colour (figure 1*d*).

**Figure 2.**
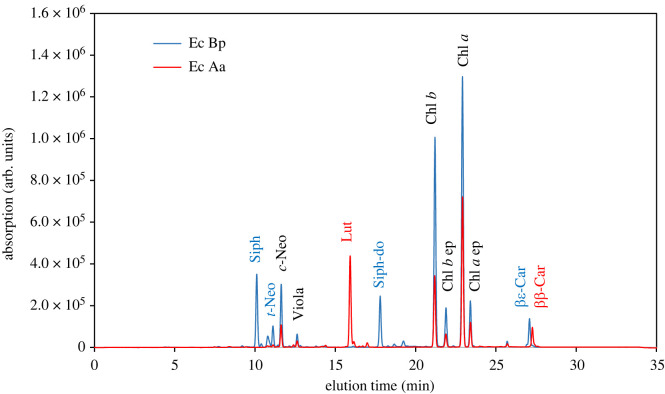
HPLC chromatograms (440 nm) showing the photosynthetic pigment composition of *E. crispata* fed with the macroalga *B. plumosa* (Ec Bp, blue line) and after 10 days feeding on the macroalga *A. acetabulum* (Ec Aa, red line). Pigments in blue are exclusive to *B. plumosa* kleptoplasts, while pigments in red are exclusive to *A. acetabulum* kleptoplasts. Pigments in black are common to the chloroplasts of both algae. Siph: siphonoxanthin; *t*-Neo: *trans*-neoxanthin; *c*-Neo: *cis*-neoxanthin; Viola: violaxanthin; Lut: lutein; Siph-do: siphonaxanthin dodecenoate; Chl *b*: chlorophyll *b*; Chl *b* ep: epimer of chlorophyll *b*; Chl *a*: chlorophyll *a*; Chl *a* ep: epimer of chlorophyll *a*; βε-Car: β,ε-carotene; ββ-Car: β,β-carotene.

Pigment analysis of *B. plumosa*-fed sea slugs revealed the photosynthetic pigments characteristic of its prey alga and of other Bryopsidales: the Chls *a* and *b*, the carotenoids siphonoxanthin and siphonaxanthin dodecenoate, *trans*- and *cis*-neoxanthin, violaxanthin and β,ε-carotene (figure 2). Pigment analysis of sea slugs that switched diet revealed that the great majority of *B. plumosa*-derived kleptoplasts were replaced by plastids of *A. acetabulum*: pigments lutein and β,β-carotene, characteristic of *A. acetabulum*, were observed, while no traces of the *B. plumosa* diagnostic pigments siphonoxanthin, siphonaxanthin dodecenoate, *trans*-neoxanthin and β,ε-carotene were found.

### 3.2. Effects of macroalgal food availability on growth and kleptoplast photosynthesis

**Figure 3.**
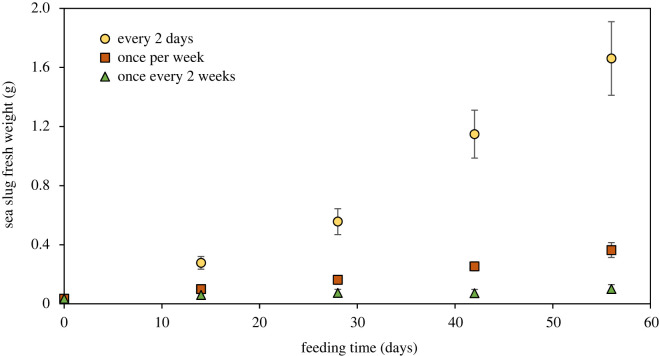
Changes in *E. crispata* fresh weight with feeding time and under different feeding conditions with the macroalga *B. plumosa*: sea slugs fed every 2 days, once per week or once every 2 weeks. Data are average ± standard deviation (*n* = 10 per treatment).

**Figure 4.**
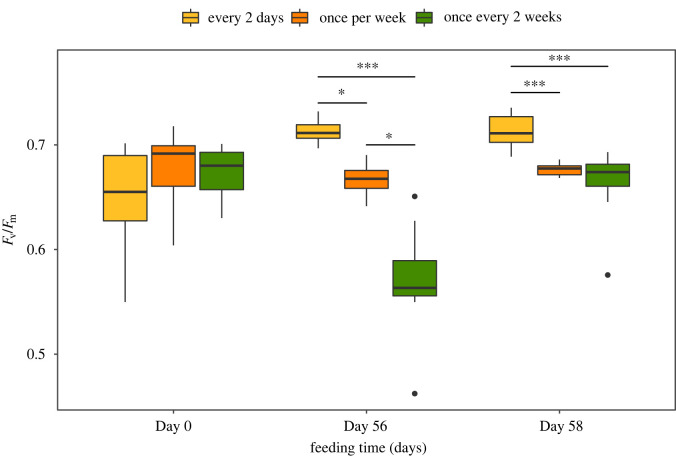
Maximum photosynthetic yield (*F*_v_/*F*_m_) of *E. crispata* under different feeding conditions with the macroalga *B. plumosa*: sea slugs fed every 2 days, once per week or once every two weeks. *F*_v_/*F*_m_ was measured at the beginning of the experiment (day 0) and at day 56, immediately before feeding. Fv/Fm was measured again 2 days after macroalgal food was provided to all the sea slugs (day 58). The line is the median, top and bottom of the box are the 75% and 25% percentiles, respectively, and the whiskers represent the maximum and minimum values (*n* = 10 per treatment). Outliers represented as black dots. * and *** indicate significant differences at *p* < 0.05 and *p* < 0.001, respectively.

Increase in sea slug weight through the feeding period, with *B. plumosa*, depended significantly on food availability (*p* < 0.001). Weight increase was more pronounced in sea slugs fed every 2 days, intermediate in animals fed once every week and lower in slugs fed once every two weeks (increase of 4598%, 940% and 181% of initial weight after 56 days, respectively) (figure 3). The diet regime showed a significant effect in the maximum photosynthetic yield of PSII (*F*_v_/*F*_m_), a measure of the photosynthetic performance of the kleptoplasts (*p* < 0.001). After 56 days, immediately before feeding, significantly higher *F*_v_/*F*_m_ was observed in sea slugs fed every two days, intermediate in animals fed once every week and lower in sea slugs fed once every two weeks (figure 4). When *F*_v_/*F*_m_ was measured on day 58, 2 days after macroalgal food was provided to the sea slugs for the last time of the feeding phase, no significant differences were observed between animals fed once every week and once every two weeks. However, *F*_v_/*F*_m_ was still significantly higher in sea slugs fed every two days.

### 3.3. Effects of previous feeding history on sea slug weight and photosynthesis during starvation

**Figure 5.**
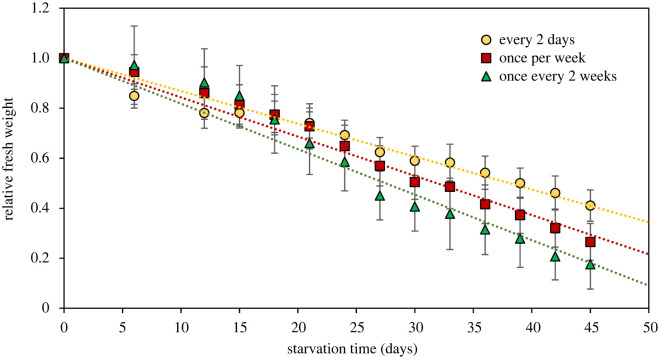
Changes in relative fresh weight of *E. crispata* under starvation, previously fed differently with the macroalga *B. plumosa*: sea slugs fed every 2 days, once per week or once every two weeks. Data are average ± standard deviation (*n* = 10 per treatment). Dotted lines represent linear regressions to the data (slopes: −0.0131, −0.0157 and −0.0182; *r*^2^: 0.9677, 0.9782, 0.9512, respectively).

**Figure 6.**
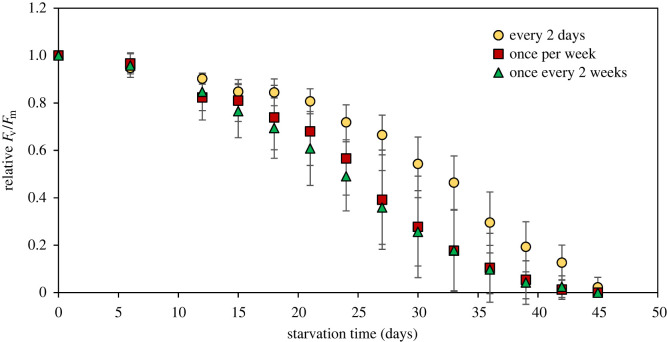
Changes in maximum photosynthetic yield (relative *F*_v_/*F*_m_) of *E. crispata* under starvation, previously fed differently with the macroalga *B. plumosa*: sea slugs fed every 2 days, once per week or once every two weeks. Data are average ± standard deviation (*n* = 10 per treatment).

When deprived of *B. plumosa* as their prey algae, sea slug fresh weight and kleptoplast photosynthetic yield decreased with starvation time (figures 5 and 6). Relative weight loss along the starvation period depended significantly on the previous food availability (*p* < 0.001): it was more pronounced in sea slugs fed once every two weeks, intermediate in animals fed once every week and lower in slugs fed every 2 days (decrease of 83%, 74% and 59% of initial weight after 45 days, respectively) (figure 5). Decrease in kleptoplast photosynthetic yield was also significantly dependent on the previous food availability (*p* < 0.001): the drop in relative *F*_v_/*F*_m_ was less pronounced in slugs fed every two days (figure 6). However, after 45 days of starvation, all sea slugs lost their photosynthetic capacity, with a residual 2% of initial *F*_v_/*F*_m_ in sea slugs fed every 2 days.

**Figure 7.**
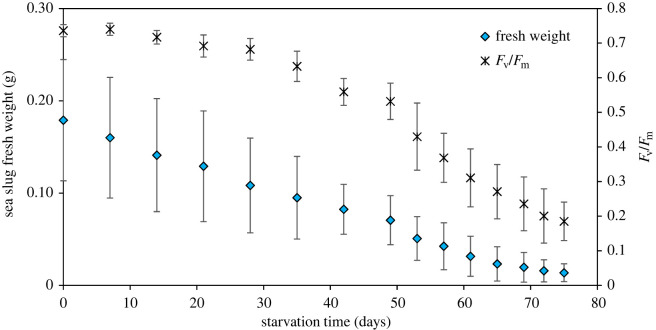
Changes in *E. crispata* fresh weight and maximum photosynthetic yield (Fv/Fm) of sea slugs under starvation, previously fed with the macroalga *A. acetabulum*. Data are average ± standard deviation (*n* = 10).

Sea slugs that switched diet from *B. plumosa* to *A. acetabulum* significantly increased their kleptoplast maximum photosynthetic yield from 0.690 ± 0.022 to 0.736 ± 0.018 (*p* = 0.004). When deprived of their prey alga, these *A. acetabulum*-fed sea slugs lost fresh weight and kleptoplast photosynthetic yield with starvation time (figure 7). However, decreases in weight and photosynthetic yield were less pronounced in these sea slugs than on animals fed with *B. plumosa*. After 49 days of starvation, relative *F*_v_/*F*_m_ was still 72% of initial photosynthetic capacity in sea slugs with kleptoplasts derived from *A. acetabulum*. About 25% of initial *F*_v_/*F*_m_ was still measurable after 75 days of starvation.

**Figure 8.**
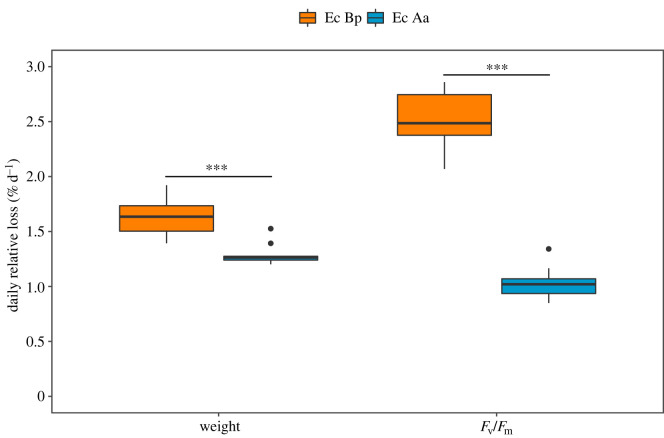
Daily relative loss of fresh weight and maximum photosynthetic yield (*F*_v_/*F*_m_) of the sea slug *E. crispata* during starvation, previously fed with the macroalgae *B. plumosa* (Ec Bp) or *A. acetabulum* (Ec Aa). The line is the median, top and bottom of the box are the 75% and 25% percentiles, respectively, and the whiskers represent the maximum and minimum values (*n* = 10 per treatment). Outliers represented as black dots. *** indicates significant differences at *p* < 0.001.

Significantly higher percentage of fresh weight loss per day was observed in sea slugs with *B. plumosa*-derived kleptoplasts than in animals with *A. acetabulum*-derived plastids (*p* < 0.001) (figure 8). Similarly, significantly higher daily loss of *F*_v_/*F*_m_ was observed in sea slugs previously fed with *B. plumosa* than with *A. acetabulum* (*p* < 0.001). Average daily loss of *F*_v_/*F*_m_ in sea slugs with *A. acetabulum*-derived kleptoplasts was 1.03 ± 0.14% d^−1^ compared with 2.52 ± 0.26% d^−1^ for animals previously fed with *B. plumosa*, indicating an over twofold longevity for the former plastids. Starved sea slugs became whitish with a distinct orange stripe along the edge of the parapodia (electronic supplementary material, figure S2).

## 4. Discussion

Larval development in *E. crispata* was lecitotrophic, with an average time to hatching of about 14 days at 25°C. In the absence of algal cues, larvae underwent intracapsular metamorphosis and hatched as crawling juveniles of about half a millimetre (figure 1*a*). Mean time to hatching of 15 days at 22°C was previously reported for *E. crispata*, with all larvae metamorphosing prior to hatching in 9 out of 11 egg masses [23]. In the two other clutches, larvae hatched as free-swimming veligers and metamorphosed within 2 days [23]. Pierce *et al*. [24] reported significantly longer encapsulation time of up to 35 days at 20°C and post-hatching metamorphosis within 5 days. Apart from natural variability between populations, differences in temperature among studies, or other incubation conditions, may explain disparities in *E. crispata* larval development [17].

Recently hatched *E. crispata* sea slugs were able to feed and acquire chloroplasts from the macroalga *B. plumosa* (figure 1*b*), but not from *A. acetabulum*. When offered the latter alga, the sea slugs did not feed on or incorporate any chloroplasts and died. A previous study analysing food choice of *E. crispata* reported that recently hatched juveniles were only able to feed on the thinner filamentous algae *B. plumosa* and *Derbesia tenuissima* out of a set of 29 algal species [25]. The authors hypothesized that the very fine filaments of the two algae could be easier for the juvenile slug to grasp and perforate to suck out the cellular content. Although filament diameter is important for food selection in sacoglossans [26,27], *B. plumosa* and *A. acetabulum* algae used in our study had similar filament thickness, indicating that this was not why recently hatched *E. crispata* were unable to feed on *A. acetabulum*. Other algal structural features, such as the cell wall composition, can influence diet selection and radular teeth morphology in sacoglossan sea slugs [28]. It is important to note, however, that cell wall calcification was not observed in the cultured *A. acetabulum*. The most likely inability of recently hatched *E. crispata* to pierce *A. acetabulum* with their radular teeth was overcome with the development of the animals, with adult sea slugs feeding on this alga and acquiring its chloroplasts.

The change in algal prey was accompanied by an alteration in the colour of the sea slugs, passing from a dark to a light green (figure 1*c,d*), as the stolen chloroplasts in the ramified digestive system changed from *B. plumosa* to *A. acetabulum* derived. Curtis *et al*. [25] reported similar differences between *E. crispata* fed exclusively on *B. plumosa* in the laboratory and wild-collected specimens. Adult *E. crispata* can feed on over 30 ulvophycean algal species belonging to the Bryopsidales and the Dasycladales [18,19,29–31]. Polymerase chain reaction (PCR)-based DNA barcoding using chloroplast encoded gene sequences (e.g. *rbc*L) has identified huge inter-population variability in terms of kleptoplast algal sources in *E. crispata* [18,19]. This molecular tool has allowed the accurate determination of the species source of the kleptoplasts in sacoglossan sea slugs, which previously used techniques (e.g. feeding experiments, electron microscopy, pigment analysis) did not permit. The most common algae indicated as the chloroplast origin of *E. crispata* belong to the genera *Bryopsis*, *Halimeda* and *Penicillus* [18,19,29]. Using DNA barcoding and more recently next-generation sequencing techniques, *Acetabularia* sp. and *Acetabularia crenulata* were identified as sources of *E. crispata* kleptoplasts, although suggested as a low-preference food source [18,30,32]. Under laboratory conditions, we show that adult *E. crispata* is clearly capable of feeding on and acquiring chloroplasts from *A. acetabulum*. In fact, the great majority of the original *Bryopsis*-derived kleptoplasts were replaced within 10 days (figure 2).

Chl *a* fluorescence measured by pulse amplitude modulated (PAM) fluorometry has been extensively used in the study of kleptoplast functionality and longevity (see review by Cruz *et al.* [14]), replacing to a large extent other methodologies such as O_2_ production (e.g. [33]) and ^14^C incorporation (e.g. [34]). Operational advantages of PAM fluorometry include real-time, rapid and non-destructive measurements of photosynthesis, allowing for multiple records over time in the same specimen. Compared with conventional optical fibre fluorometers that provide a measurement in a pre-chosen spot of the sea slug body, imaging PAM systems have the advantage of allowing the measurement of the entire animal (electronic supplementary material, figure S1). The main Chl *a* fluorescence parameter used in the study of photosynthetic activity in sea slugs has been the maximum quantum yield of PSII (*F*_v_/*F*_m_), providing a general evaluation of the health status of the stolen chloroplasts [4,14].

*Bryopsis plumosa* availability during the feeding period had a significant effect on the reduction of *F*_v_/*F*_m_ throughout starvation (figure 6). This indicates that continuously fed sea slugs constantly replace the older kleptoplasts, initiating the starvation period with a set of chloroplasts with higher photosynthetic capacity (figure 4). However, after 45 days of starvation, *F*_v_/*F*_m_ reached close to zero values in all three feeding treatments, indicating this period as the kleptoplasts' maximum longevity under the specific experimental conditions used. On the other hand, significantly higher *F*_v_/*F*_m_ throughout starvation was observed in sea slugs previously fed on *A. acetabulum* than on *B. plumosa* (figures 7 and 8). The chloroplasts stolen from *A. acetabulum* were still photosynthetically active in *E. crispata* after 75 days of starvation, showing that the origin of the plastids is extremely relevant to the longevity of the association. In a previous study, *F*_v_/*F*_m_ in starved *E. crispata* previously fed on *B. plumosa* was shown to decrease linearly to zero in 10 weeks, while in *P. lamouroxii*-fed specimens *F*_v_/*F*_m_ was still measurable after 12 weeks of starvation [8]. Different culturing conditions, including the higher irradiance used, may explain the shorter longevity of *B. plumosa* kleptoplasts reported in our study.

Under natural fluctuating light conditions, the presence of more efficient protection mechanisms in *A. acetabulum* chloroplasts may explain why these plastids are maintained functional for longer periods in *E. crispata* than *Bryopsis*-derived kleptoplasts. *Acetabularia acetabulum* chloroplasts have a fully active xanthophyll cycle, comprising the sequential de-epoxidation of the pigments violaxanthin to antheraxanthin and zeaxanthin, which ultimately leads to the dissipation of excessive light energy [35]. This ubiquitous photoprotection mechanism, present in plants and algae, is absent in many Bryopsidales, including *B. plumosa* [36,37]. Moreover, the chloroplasts of *A. acetabulum*, as well as the kleptoplasts in *E. timida*, have been shown to use oxygen-dependent alternative electron sinks (e.g. flavodiiron proteins) from photosystem I, maintaining it oxidized and less prone to photoinhibition [20]. Flavodiiron proteins are common in green algae, but their existence and functionality in *Bryopsis* remain to be tested [38].

The above-mentioned photoprotection mechanisms are expected to function mainly under higher light conditions [39], so it is questionable if they play a role in kleptoplast longevity in the relatively low and stable light settings used in our study. On the other hand, photoinhibition of PSII is known to take place whenever PSII is excited by photons, and this damage needs to be constantly repaired [40]. Under the studied light conditions, it is therefore more likely that the main differences in the longevity of kleptoplasts derived from *A. acetabulum* and *B. plumosa* relate to their innate capacity to repair PSII and the general oxidative damage caused to the kleptoplasts during photosynthesis. Available reports on the capacity of *A. acetabulum* chloroplasts to maintain PSII repair cycle are somewhat contradictory. While some studies have suggested that the alga *A. acetabulum* itself is quite efficient in repairing PSII after photoinhibition [41] and that this repair capacity is still largely working in the kleptoplasts in *E. timida* [42], others suggest that *A. acetabulum* kleptoplasts in *E. timida* do not carry out significant PSII repair [43]. In their study, Christa *et al*. [43] also concluded that the *Bryopsis hypnoides* kleptoplasts in the sea slug *Elysia viridis* are similarly defunct when it comes to PSII repair cycle. However, what is true to all the aforementioned studies on PSII repair cycle in kleptoplasts is that none of them examined the repair cycle on a protein level, i.e. by assessing the degradation and re-synthesis of the D1 protein of PSII, which is at the very core of PSII repair cycle. The capacity for photosynthetic sea slugs to avoid or repair oxidative damage in the stolen chloroplasts is probably highly important for the maintenance of long-term functional kleptoplasts, but more in-depth studies at the molecular level are needed to fully solve the true extent of that importance [9].

Sacoglossan sea slugs that harbour long-term functional chloroplasts have wing-like parapodia. In some species, like *E. timida* and *P. ocellatus*, closure of the parapodia have been shown to shield the kleptoplasts from high irradiance [35,44]. In these sea slugs, the kleptoplasts are distributed on the inner part of the parapodia, so their closure can efficiently reduce irradiance reaching the kleptoplasts and alleviate photodamage. The efficiency of such behavioural mechanism in *E. crispata* is questionable due to the wavy morphology of the parapodia and the visible chloroplast-containing digestive diverticula on the external surfaces.

More regular feeding on *B. plumosa* resulted in sea slugs with significantly higher body mass, showing that heterotrophic nutrition is decisive in determining animal size (figure 3). However, it is difficult to differentiate between autotrophic and heterotrophic pathways, as food availability also affects photosynthesis because of the pace at which older chloroplasts are replaced. Indeed, photosynthetic activity decreased in intermittently fed sea slugs (figure 4), showing that food availability has also an impact on the autotrophic pathway. Higher *B. plumosa* availability during feeding resulted in a slower relative decrease in weight once sea slug specimens were starved (figure 5). On the other hand, significantly lower relative weight loss under starvation was observed in sea slugs fed on *A. acetabulum* than on *B. plumosa* (figure 8). The latter result suggests a relevant role of photosynthesis of the higher-performance chloroplasts of *A. acetabulum* to the nutrition of *E. crispata*. Previous studies have shown the relevance of kleptoplast photosynthesis in minimizing weight loss and increasing survival in periods of food scarcity in sea slugs such as *E. viridis*, *E. timida*, *E. chlorotica*, and *P. ocellatus* [45–48]. It has recently been suggested that kleptoplast photosynthesis may further potentiate sacoglossan sea slugs’ evolutionary success by maximizing its reproductive output [49].

## 5. Conclusion

This study shows that functionality and longevity of kleptoplasts in a polyphagous kleptoplastic sea slug depend on the origin of the plastids. However, different sea slug species feeding and acquiring chloroplasts from the same macroalga show different capacities to maintain the kleptoplasts functional [5–7]. Hence, the functionality of the stolen chloroplasts in sacoglossan sea slugs must result from a combination of inherent robustness of the original chloroplasts with host cell metabolic attributes that potentiate the longevity of the association. We now know of a common prey alga, *A. acetabulum*, which can be used to specifically inspect the differences between monophagous short-term retention and long-term retention sea slugs *E. cornigera* and *E. timida*, as well as between monophagous and polyphagous long-term retention sea slugs *E. timida* and *E. crispata*. This opens new experimental possibilities to crack the puzzle of kleptoplasty in photosynthetic sea slugs.

## Data Availability

Data submitted as electronic supplementary material (electronic supplementary material, data S1) [50]. File name: electronic supplementary material, data S1. Raw data of Prey species and abundance affect growth and photosynthetic performance of the polyphagous sea slug *Elysia crispata*.
